# Control of the Lung Residence Time of Highly Permeable Molecules after Nebulization: Example of the Fluoroquinolones

**DOI:** 10.3390/pharmaceutics12040387

**Published:** 2020-04-23

**Authors:** Julien Brillault, Frédéric Tewes

**Affiliations:** 1 INSERM U-1070, Pôle Biologie Santé, 86000 Poitiers, France; 2 UFR Médecine-Pharmacie, Université de Poitiers, 86073 Poitiers, France

**Keywords:** pulmonary administration, fluoroquinolones, controlled release, lung permeability, metal complexes, microparticles, liposomes, nanoparticles, residence time, biopharmaceutical

## Abstract

Pulmonary drug delivery is a promising strategy to treat lung infectious disease as it allows for a high local drug concentration and low systemic side effects. This is particularly true for low-permeability drugs, such as tobramycin or colistin, that penetrate the lung at a low rate after systemic administration and greatly benefit from lung administration in terms of the local drug concentration. However, for relatively high-permeable drugs, such as fluoroquinolones (FQs), the rate of absorption is so high that the pulmonary administration has no therapeutic advantage compared to systemic or oral administration. Formulation strategies have thus been developed to decrease the absorption rate and increase FQs’ residence time in the lung after inhalation. In the present review, some of these strategies, which generally consist of either decreasing the lung epithelium permeability or decreasing the release rate of FQs into the epithelial lining fluid after lung deposition, are presented in regards to their clinical aspects.

## 1. Introduction

Pulmonary delivery of drugs is an interesting alternative to oral absorption. Due to its high surface of exchange, the thickness of the alveolar wall, and the rich capillary network, lung absorption is expected to be fast and lead to a rapid onset of the systemic drug’s action. The other advantages are low local metabolic activity and no first bypass metabolism [1]. However, in the case where the lungs are the site of drug action, such as in pulmonary infections, a low rate of absorption of antibiotics is preferred. This can lead to a higher local concentration of antibiotics, better treatment outcomes, and reduced systemic side effects compared to systemic administration [2]. The alveolar surface represents the main absorption surface of the lungs (≈ 100 m² compared to 2 m² for the conductive airways). The first component of the pulmonary epithelial barrier that faces a drug after its pulmonary deposition is the thin fluid lining the epithelium. The composition of this epithelial lining fluid (ELF) changes in the different pulmonary functional zones. In the conducting zone, it is made of a mucus layer, and in the respiratory zone, it is mainly composed of a watery layer and surfactant, with a small total volume of 10–40 mL distributed over a large area of 35–100 m² [3]. Hence, the water solubility of the inhaled drugs is a significant challenge for dissolution, especially for anti-infective drugs that are administered in high doses to be effective (≈ 10–30 mg) [4]. However, unlike what happens in the intestine, where undissolved drugs are eliminated through the gastrointestinal transit, the alveolar space is a dead-end with only macrophages’ phagocytosis and absorption as elimination processes. As a result, most particles have a higher average residence time in the lungs compared to what they would have in the intestine [5]. Therefore, particles that have a slow drug dissolution rate or a slow drug release rate relative to the drug absorption rate can be advantageously used as a strategy to achieve a stable drug concentration in pulmonary ELF [5]. This approach is strengthened when the particles can escape phagocytosis from macrophages, such as for PEGylated particles or for large porous particles with a geometrical diameter larger than 6 µm but an aerodynamic diameter lower than 5 µm [6]. Hence, the apparent drug water solubility should be finely tuned through formulation to balance the controlled dissolution/release and drug efficiency in the ELF. In a second time, the deposited compound encounters the lung epithelium, whose composition also changes along the respiratory tract. In the conducting zone, it is a thick epithelial layer comprising several types of cells, mainly ciliated, which play an important role in the mucociliary clearance. In the respiratory zone, the epithelium is mainly composed of thin alveolar type I cells (0.1–0.5 µm). The drug permeability of this epithelium is mainly governed by passive diffusion and therefore by the molecular weight and lipophilic properties of the drug. For instance, hydrophilic molecules, such as aztreonam, colistin, or tobramycin, have been evaluated as low apparent permeability drugs (apparent permeability (P_app_) < 0.1 × 10^−6^ cm/s) in in vitro pulmonary models of drug transport using Calu-3 cells [7,8,9,10]. When administrated to rats by intratracheal (IT) nebulization, a low absorption rate and high ELF/plasma area under the concentration–time curve (AUC) ratios were observed (1214, 1069, and 230 for colistin, aztreonam, and tobramycin, respectively). However, in similar studies, lipophilic fluoroquinolones (FQs), such as ciprofloxacin (CIP) and moxifloxacin, were defined as high-permeability drugs for pulmonary absorption, with a P_app_ > 1 × 10^−6^ cm/s. After IT nebulization, these FQs’ lung PK profiles showed a rapid equilibrium between the lung and plasma concentrations. Hence, almost similar PK profiles in plasma and ELF were obtained regardless of the route of administration, showing no advantage of FQ nebulization over intravenous (IV) administration [11]. The effect of low permeability on the pulmonary bioavailability of a drug in the lungs is one of the reasons for the development of inhaled antibiotic formulations, such as tobramycin (TOBI^®^, TOBI^®^ Podhaler), aztreonam (Cayston^®^), and colistin (Colobreathe^®^). However, in the case of FQs, the high permeability and rapid absorption through the lung is an issue that may lead to sub-therapeutic concentrations at the site of infection after inhalation, which can lead to treatment failure and bacterial resistance development [11,12,13,14]. Yet, quinolones are a very important class of antibiotics, rich in more than 30 members, with broad-spectrum activities, and have been widely used as therapy for respiratory infection. The available quinolones are mostly FQs and include CIP, gemifloxacin, levofloxacin (LVX), moxifloxacine, norfloxacine, and ofloxacin. Other FQS were introduced and then withdrawn due to severe adverse effects, such as hepatotoxicity (temafloxain, gatifloxacin, trovafloxacin), or severe cardiovascular events (grepafloxacin) [15]. From the perspective of pulmonary delivery, adverse effects are expected to be significantly reduced and several FQs could be re-evaluated for this way of administration as long as the low residence time issue is resolved. There are several ways to increase the FQ lung residence time after inhalation, such as lowering their P_app_ through the lungs or controlling their release rate from formulations. In this sense, metal cation complexes, liposomal formulations, dry-powder microparticles, and lipid nanocarriers have received much interest in recent years as promising technologies for the administration of FQs by inhalation [16,17,18,19,20,21,22,23,24,25]. In the following sections, these two aspects—the control of dissolution rate and the control of permeability—will be discussed with regard to obtaining high and stable concentrations of drugs in the lungs after the inhalation of molecules with high permeability, such as FQs.

## 2. Control of FQ Lung–Blood Barrier Permeability by Pulmonary Administration of FQ–Metal Complex

Most FQs complex metal multivalent cations to form positively charged chemical species that have increased aqueous solubility and decreased permeability across the biological barrier compared to free FQs [26]. As a consequence, the complexation of CIP with cations, such as calcium, ferrous iron, or aluminum, reduces its relative oral bioavailability by 40%, 50%, and 85%, respectively [27,28]. A strong correlation between the reduction in the oral bioavailability of FQs in the presence of divalent and trivalent cations and the magnitude of complexation constants measured in vitro has often been established [29,30]. This drawback for the oral route of administration of FQs can be turned into an advantage for their pulmonary delivery as aerosols in order to increase their residence time in the lung.

### 2.1. Metal Cation–FQ Complexes to Control FQs’ Permeability across the Lung–Blood Barrier

Complexes formed between FQs and metal multivalent cations are positively charged and thus have decreased permeability across biological barriers compared to free forms. For instance, CIP is an overall uncharged zwitterionic molecule at pH 7, but in the case of a 1:1 CIP^+/−^: metal^2+^ cation interaction stoichiometry, a bicationic complex CIP^+^-M^+^ is formed, reducing its apparent solubility into the phospholipid cell membrane and its P_app_, while increasing its water solubility due to the ion interactions with water molecules. Numerous metal cations were shown to bind to FQs, such as Ca^2+^, Mn^2+^, Mg^2+^, Zn^2+^, Fe^2+^, Fe^3+^, Al^3+^, Cu^2+^, Ag^+^, Co^2+^, Ni^2+^, Bi^3+^, Au^3+^, etc. There are two main sites involved with the metal interaction on FQ molecules: One is constituted by the carbonyl and carboxyl groups on the quinolone ring and the second implies the piperazin group [26]. FQ–metal cation chelates may be synthetized as 1:1, 2:1, or 3:1 (FQ:metal cation) complexes depending on the FQ, cation, pH, and synthesis process. In solution, they may spontaneously form as 1:1 or 2:1 complexes. It is important to note that not all metal cations have the same affinity for FQs. For instance, CI–metal’s apparent constant of association, determined in a simple saline buffer (phosphate buffered saline (PBS), pH 7.4), varies within a range of approximately 1 to 10,000 between Ca^2+^ and Cu^2+^ (Table 1). FQs–metal cation association constants usually rank in the same order, where the highest interactions are obtained with Cu^2+^, Al^3+^, or Fe^3+^ while the lowest are obtained with Ca^2+^ or Mg^2+^ (Table 2). In terms of permeability and solubility, the consequences on drug bioavailability after oral administration are well known: Complexation of CIP with Ca^2+^, Fe^2+^, or Al^3+^ reduces its relative oral absorption.

**Figure 1:**
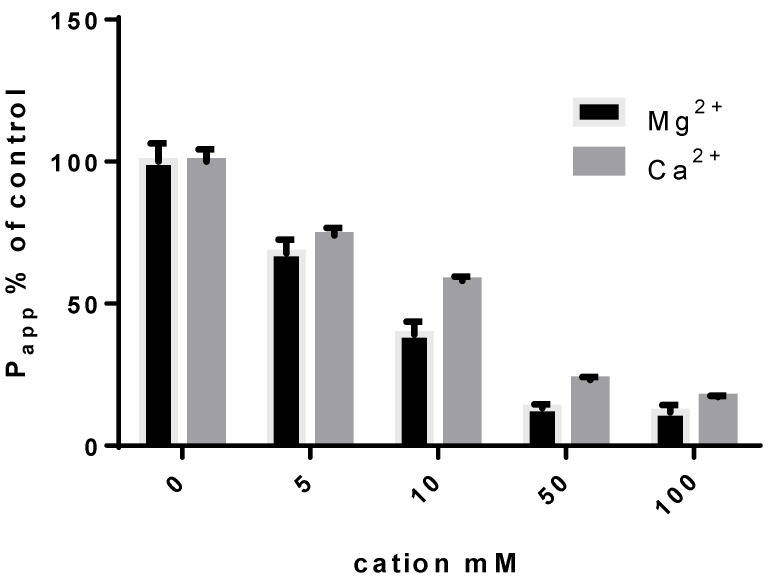
Influence of the Mg^2+^ or Ca^2+^ concentration on the ciprofloxacin (CIP) P_app_ across the lung epithelial cell monolayer (Calu-3 cells) [23,31]. The apparent permeability was evaluated in the apical-to-basal direction. The same 50 µM concentration of CIP was used in each condition, in the presence of different concentrations of Ca^2+^ or Mg^2+^.

This limitation for the oral route can be turned into an advantage for pulmonary delivery as aerosols in order to decrease their absorption from the lung. As an example, in vitro experiments using Calu-3 cells as a model of the lung epithelium have showed that increasing concentrations of calcium or magnesium dramatically decreased the apparent permeability (P_app_) of CIP (Figure 1). These results were also supported by in vivo experiments where inhalation of CIP–Ca^2+^ or CIP–Cu^2+^ complex-loaded microparticles in rat lungs succeeded in a higher CIP ELF concentration than with a CIP solution [22]. The apparent association constant is useful to predict the effect of the FQ–metal interaction on the cell permeability. For instance, the association constant values from Table 1 were used to calculate the concentration of cations necessary to reach 80% of the CIP complexation (i.e., Ca^2+^: 40 mM, Mg^2+^: 5.5 mM, Zn^2+^: 1.5 mM, Al^3+^: 0.1 mM and Cu^2+^: 0.04 mM, for a 50 µM CIP concentration). This 80% of complexations between CIP and the different ions led to a 50% decrease of P_app_ in the in vitro Calu-3 lung epithelium model whatever the nature of the cation. The difference between Ka values has huge consequences when regardless of the pulmonary formulation. For instance, if a formulation is designed to deliver in the ELF 1 μg/mL (3 μM) of CIP, which is in the range of the minimum inhibitory concentration (MIC) for the majority of *Pseudomonas aeruginosa* (*PA*) [35], the ion concentration in the ELF necessary to reach 80% of complexation would be 1600 μg/mL for Ca^2+^ (Ka ≈ 100 M^−1^) and only 0.4 μg/mL for Cu^2+^ (Ka ≈ 900,000 M^−1^) [31]. This has to be taken into consideration as the administrable amount of powder is limited for the patient’s comfort to approximatively 100 mg, including excipients in several inhalations (e.g., TOBI^®^ Podhaler^®^ uses capsules with 55 mg of powder, of which 28 mg of tobramycin, for a total dose of 4 capsules) [36]. Hence, metal complexation is a promising strategy to lower the lung permeability of FQs for inhaled therapies, and recent formulations with CIP or LVX are actually available in the market or in development.

### 2.2. Quinsair^®^/Aeroquin^™^: A Solution for Pulmonary Inhalation of the Fluoroquinolone–Metal Complex Marketed

The main advantage of administering a solution of FQ–metal complex over a controlled release system is that once in the lungs, all FQ molecules will be immediately available to produce their effect. Indeed, most of the FQ–metal complexes have a similar or higher antibacterial activity than the free form [26]. This is particularly interesting for patients colonized by a bacterial strain with a high MIC or to eliminate a resistant bacterial subpopulation. Treatment should be more effective if there is a high loading dose to more effectively kill pathogens with high MICs in the lungs. This is also particularly true for antibiotics, such as FQs, which are considered to be concentration-dependent antibiotics.

#### 2.2.1. Pharmaceutical Properties—Development

The inhaled LVX aqueous solution initially called MP-376 (brand name Quinsair^®^ in Europe, or Aeroquin™ in the USA) is a FQ formulation originally developed for the long-term treatment of lung infections caused by *PA* in cystic fibrosis (CF) patients [32,37,38,39,40,41,42,43]. This formulation is the first FQ-inhaled formulation approved in the European Union and Canada. Requests for approval in the United States have been made. It is approved for the treatment of chronic pulmonary infections due to *PA* in adults with CF but has also been evaluated for the treatment of *PA* chronic infections in patients with non-CF bronchiectasis (NCFB) and in patients with non-tuberculous mycobacteria.

The commercialized form of MP-376 is formulated for aerosol administration of 240 mg of LVX (246 mg of LVX hemihydrate) solubilized in 2.4 mL of a preservative-free, 350–500 mOsmol, pH 5–7, aqueous solution, which is administered using a customized eFlow^®^ vibrating membrane nebulizer (PARI Pharma, Munich, Germany) [38,40]. In this formulation, LVX is complexed with Mg^2+^. In the articles [42,44] and reports [44] published on this formulation, the complexation with Mg^2+^ is described as being used to increase the aqueous solubility of LVX and to mask its bitter taste.

In patents [14,25,26,28,32], which support the development of this formulation, the initial studies started with the selection of the FQ that was going to be used. To do this, they evaluated 13 FQs that had already been marketed according to five criteria: Anti-*PA* activity, ability to make a solution at 10 mg/mL, pH adjustment needed to solubilize the molecules, aerosol tolerability in healthy volunteers (cough and cough sensation), and taste. Aerosolized solutions with pH below 4.5 are poorly tolerated. Thus, the FQs that required a pH lower than 4.5 to be solubilized at 10 mg/mL, such as the CIP, were rejected. LVX, ofloxacin, and moxifloxacin exhibited the best solubility/pH characteristics [32]. LVX, which was the most tolerable FQ at the concentration tested, and has one of the best antimicrobial profiles against *PA* (comparable to CIP), was thus selected. At this stage of development, the main limitation of the LVX solution was its bitterness. Various excipients have been tested to mask this taste. Among these, the use of divalent metal cations (Mg^2+^, Ca^2+^, Zn^2+^, Fe^2+^, Al^3+^), known to complex the FQ, were further assessed. The main result presented in the patent showed that the complexation of LVX with Mg^2+^ reduced the bitterness, and increased up to 3 times its apparent aqueous solubility [32]. At the pH of the ampoule (between 5 and 7), LVX solubility can reach values close to its concentration in the formulation. Consequently, the control of the aqueous solubility of LVX was considered to be important in the development of this formulation [44] and could be one of the reasons why divalent metal salts were chosen to mask the bitterness of the solution among the other excipients tested [32].

The increase in aqueous solubility resulting from complexation with metal cations is due to the formation of positively charged species, which are more polar than the free LVX. This increase in polarity also reduces its P_app_ across biological barriers compared to free LVX. In the patents, the kinetic profiles are presented, consisting of the quantities of LVX found in the lungs of rats after nebulization of LVX at 10 mg/kg in the form of saline solution or formulated with various divalent cations (Ca^2+^, Mg^2+^, or Zn^2+^) [25]. This amount increased with the presence of cations and with the association constant of the LVX–cation complexes. The complexed forms showed slower lung clearance to the plasma than for free LVX. In rats, the nebulization of LVX formulated with Ca^2+^ or Mg^2+^ produced a 2- to 5-fold higher LVX C_max_ and the area under the concentration–time curve (AUC) in bronchial alveolar lavage (BAL) compared to intravenous LVX or aerosolized LVX formulated in saline [25]. The pharmacokinetics (PK) of MP-376, which contains Mg^2+^, have been characterized in a mouse model of pulmonary infection [45]. The authors reported that aerosolized administration achieved a 9-fold higher AUC and 30-fold higher maximum C_max_ in lung tissue when compared with the dose-normalized intraperitoneal administration of LVX. Similar results were shown in CF patients [25].

#### 2.2.2. Pharmacokinetics—Clinical Studies

From 2007 to 2014, nine clinical studies were conducted with MP-376. Seven of these nine studies included patients with CF and two included chronic obstructive pulmonary disease (COPD) patients [44]. Four studies (MPEX-202, 203, 205, and 206) were mainly designed to assess the safety, tolerability, and PK of MP-376. In most of the studies, an antacid containing Al(OH)_3_ and Mg(OH)_2_ was administered orally prior to and after each inhaled dose to minimize oral absorption. Thus, the LVX serum concentrations mainly reflected absorption from the airways. In a preliminary study, single aerosolized doses of two dose levels (20 and 40 mg—respirable delivered dose) of LVX for parenteral use (Levaquin^®^), not MP-376, were given to CF patients and healthy volunteers with an eFlow^®^ nebulizer (PARI Pharma, Munich Germany) [25,44]. The main finding was that absorption of LVX occurred significantly slower in patients with CF than in healthy patients, and 50% of the dose remained in the lungs for at least 0.5 h after dosing. Thus, the AUCs of sputum in CF patients were 70 times higher after nebulization of 40 mg of LVX than after the IV dose of 50 mg. However, sputum concentrations still tended to drop rapidly during the first 2 h of administration, consistent with quick drug absorption from the lungs.

In study MPEX-202, the bioavailability of LVX in healthy volunteers was compared to that in patients with CF after single ascending inhaled doses of MP-376. This study showed that the LVX exposures were much higher in the sputum than in the serum in patients with CF (median C_max_ estimates were 100–250 higher and AUC estimations were 40–200 higher in the sputum than in the serum). In MPEX-203, multiple doses of MP-376 were nebulized in patients with CF up to twice a day for 14 days. Mean serum LVX concentrations increased for all doses tested between day 1 and day 15. Again, exposure of LVX in the sputum was higher than in the serum (70 to 1000 times) [44].

The MPEX-205 study concerns the nebulization of MP-376 to CF patients receiving a single 180 mg dose of LVX using 50 or 100 mg/mL solutions, followed by 7 days of daily treatment with a single dose of 240 mg using 100 mg/mL solutions [38,44]. LVX plasma PK after single- and multiple-dose administration of the MP-376 formulation at 100 mg/mL was linear (independent of the dose). The two LVX concentrations used to get the 180 mg dose gave high sputum concentrations (sputum C_max_ of 2563 and 2932 mg/L, for 50 and 100 mg/mL, respectively) associated with low systemic concentrations (serum C_max_ of 0.95 and 1.28 mg/L, for 50 and 100 mg/mL, respectively). The 240 mg dose gave higher sputum concentrations (C_max_ of 4691 mg/L), still with low systemic concentrations (serum C_max_ of 1.72 mg/L). However, the standard deviations were too high to conclude that the sputum exposure to LVX was proportional to the dose. In the phase 2 and phase 3 studies (MPEX-204, MPEX-207, and MPEX-209), sparse PK sampling and population PK were used to estimate serum and sputum LVX exposures at steady state. A simple four-compartmental model was used to fit both the serum and sputum data. The estimates of LVX C_max_ and AUC_0-24_ in the sputum expressed as a number of times those were calculated in the serum are summarized in Table 3.

A comparison of the distribution of steady-state AUC_(0-24)_ between MPEX-207 and MPEX-209 with the patients in MPEX-204 showed significant overlap in the distributions of PK exposure parameters between studies. Based on these studies, the average systemic exposure of the tested population to LVX after the administration of MP-376 240 mg twice daily (BID) was about 70% of that obtained after administration of 250 mg of LVX orally. Inhalational administration of MP-376 resulted in LVX exposures in the lung of CF patients, based on sputum concentrations, which were generally more than 100 times greater than those obtainable after the administration of ofloxacin orally (50% of which is LVX) [44].

#### 2.2.3. Efficacy

LVX possesses a potent activity against *PA*, which, in contrast to tobramycin, is not affected by the presence of CF patients’ sputum [32,46,47]. The MP-376 formulation contains Mg^2+^, which complexes LVX, increasing its apparent solubility, and decreasing its bitterness and P_app_ across the biological barrier. However, in some published works, Mg^2+^ is associated with a reduction in the potency of some FQs [26]. In vitro comparisons of LVX formulated in saline or MP-376 showed similar activity against various *PA* strains [44]. In animal models, the extent of bacterial killing of resistant strains of *PA* in single- and repeated-dose models was greater with IT administration (administered at the bifurcation of the trachea, which provides 100% delivery to the lungs) than with intraperitoneal (IP) administration. In addition, inhalation doses of MP-376 had better activity than LVX in saline against resistant strains of *PA*. LVX at 60 mg/kg was equally or more active than aztreonam (400 mg/kg) and tobramycin (60 mg/kg) against several strains of *PA* after inhalation in mice [45].

The main clinical efficacy data come from the MPEX-204, 207, and 209 phase 2 and 3 studies. In MPEX-204, the efficacy of MP-376 was evaluated among 151 patients with stable CF and evidence of chronic *PA* lung infection [40,44,48]. Participants were randomized to one of three MP-376 dose regimens (240 mg BID, 240 mg once daily (QD), 120 mg QD), or placebo for 28 days. The primary efficacy endpoint was the change in sputum *PA* density. The 240 mg BID group had the greatest reduction in *PA* density at day 28, with a −1.19 (log CFU/g sputum) difference vs. the placebo. The MPEX-209 study compared MP-376 to a tobramycin solution for inhalation (TIS) in people with CF who had chronic *PA* lung infection [37,41]. This study showed that LVX was not inferior to TIS as measured by lung function. The adverse event profile was similar for both MP-376 and TIS groups; however, MP-376 treated participants complained more frequently about the taste of the medication [37,44,49]. A systematic literature review and Bayesian network meta-analysis was conducted to compare the relative short-term (4 weeks) and long-term (24 weeks) outcomes of the commercialized inhaled antibiotics (tobramycin, colistimethate sodium, aztreonam) versus MP-376 [50]. At 24 weeks, none of the treatments were significantly more effective than MP-376.

#### 2.2.4. Safety

Two trials have investigated the safety of the inhaled LVX solution MP-376 in CF patients. Overall, due to the significant systemic exposures to LVX observed after 28-day inhalation cycles of MP-376, the same toxicological profile as that defined for systemic LVX arises with long-term use of MP-376 [44].

### 2.3. Dry Powder of Fluoroquinolone–Metal Complexes for Better Patient Compliance

The antibiotic solution nebulization treatment usually lasts about 20 min and up to 45 min, once or twice a day [51,52]. This extended daily treatment method can have a negative impact on treatment compliance. Dry powder inhalation is much more convenient than nebulization for chronic treatment as it considerably reduces the time required for the administration of a dose. In addition, nebulizers can more easily be contaminated when not properly cleaned compared to dry powder inhalers (DPIs) [51,52]. Hence, the antibiotics that were first formulated as a solution for inhalation were then developed as dry powders. For instance, tobramycin was approved by the Food and Drug Administration (FDA) in 2013 to treat lung infection in CF patients via a DPI (TOBI^®^ Podhaler^®^) and a colistimethate sodium DPI (Colobreathe^®^) received European approval in 2012. Thus, an attempt has recently been made to develop a dry powder form of an FQ–metal complex [22,23,24]. Such a dry powder form of an FQ–metal complex could provide an opportunity to combine both the controlled release approach and the controlled permeability approach to further extend the residence time of an FQ in the lung.

#### 2.3.1. Pharmaceutical Properties—Development

In order to take advantage of the reduction in the P_app_ of CIP through the pulmonary epithelium in the presence of calcium, inhalable calcium-based microparticles have been developed to increase the residence time of CIP in the lungs after inhalation in the form of a dry powder [23].

These composite microparticles, formulated by spray drying, consist of hyaluronate (a biopolymer) and mineral components comprising amorphous calcium carbonate and amorphous calcium formate [23,53]. Cupric ion (Cu^2+^) complexes CIP with 10,000-fold more affinity than calcium ion. As a result, 10,000-fold less Cu^2+^ than Ca^2+^ is needed to reduce the CIP apparent permeability by 50% across a pulmonary epithelium model [31]. To evaluate the in vivo effect of this control of the CIP P_app_ observed in vitro, microparticles loaded with CIP–copper complex were developed [22] and recently patented [54]. Due to their amorphous nature and small size, these particles are highly soluble in artificial lysosomal fluid and simulated lung fluid. These shell-like particles have aerodynamic properties suitable for topical respiratory treatment but have limited fine particle fraction (38.6%) [23]. To improve their aerodynamic properties, the particles have been reformulated using L-leucine, known for its anti-adherent properties, to increase the dispersibility of the powder [24]. The addition of L-leucine allowed the production of particles with a median geometric and aerodynamic diameter of 3.2 and 3.4 μm, respectively. The fine particle fraction of the powder emitted from a Handihaler^®^ dry powder device was increased up to 65.4%, predicting high total lung deposition.

#### 2.3.2. Pharmacokinetics

**Figure 2:**
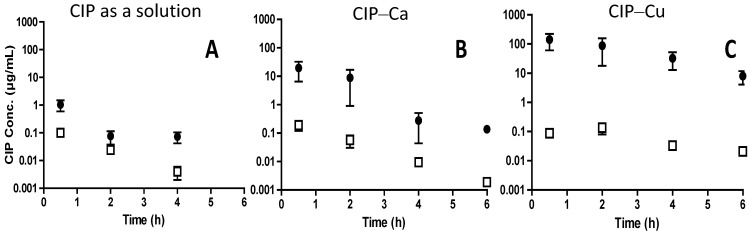
CIP concentration profile in the lung epithelial lining fluid (ELF) (plain dot) and free CIP plasma concentration profile (open square) obtained after IT administration to rats of (**A**) a CIP solution, (**B**) particles loaded with the CIP–Ca low-affinity complex, or (**C**) particles loaded with the high-affinity CIP–Cu complex. The CIP dose was 3 mg/kg. Each point is an averaged value ± standard deviation (SD) of 4–7 individual measurements (adapted from Lamy et al. [22]).

PK studies have been conducted in healthy rats [22] and in model rats with chronic lung infection [55]. In healthy animals, CIP (3 mg/kg) was delivered IT as a solution using a MicroSprayer IA-1B apparatus (Penn-Century Inc, Philadelphia, USA) or as microparticles loaded with either the low-affinity CIP–calcium complex (CIP–Ca) or with the high-affinity CIP–copper complex (CIP–Cu) using a Dry Powder Insufflator (Penn-Century Inc, Philadelphia, USA). These two administration systems directly deliver the formulations to the carina of the trachea (IT administration), which provides 100% delivery to the lungs. At predetermined times, a bronchoalveolar lavage (BAL) and a blood sample were taken to measure the CIP plasma and ELF concentrations (Figure 2). For the CIP solution and CIP–Ca microparticle formulations, CIP ELF concentrations decreased in parallel with CIP plasma concentrations and the mean t_1/2_ in ELF was similar to that measured in plasma (0.82 h). In comparison, higher mean t_1/2_ in ELF (2.1 h) and higher CIP ELF concentrations were measured after CIP–Cu microparticles’ IT administration (Figure 2). Accordingly, the CIP AUC_ELF_ to AUC_,plasma_ ratios were equal to 1069, 203, and 9.8 after CIP–Cu microparticles, CIP–Ca microparticles, and CIP solution pulmonary administration, respectively (Table 4). Based on these ratio values, CIP lung exposure obtained with the CIP–Cu microparticles was 100-fold and 5-fold higher than the exposures obtained by the administration of a CIP solution or CIP–Ca microparticles, respectively.

Besides, CIP plasma t_1/2_ was also increased after the pulmonary administration of CIP–Cu microparticles (3.2 h) compared to the other formulations (0.82 h). This suggests that the rate of absorption of CIP from the lung to the blood was reduced enough to become slower than intrinsic CIP plasma t_1/2_ and thus became the limiting step controlling the elimination of CIP from the plasma. This phenomenon is described as flip-flop kinetics, because the rate of drug elimination is limited due to the slow rate of absorption [56].

A similar protocol was used to perform PK on rats with chronic PA01 strain lung infection. On day 8 post-infection, a group was treated IV with a CIP solution and another received the CIP–Cu-loaded microparticles IT [55]. A higher CIP AUC_ELF_ to AUC_plasma_ ratio was obtained after the pulmonary administration of CIP–Cu microparticles to infected animals (3560) compared to healthy rats (1069).

#### 2.3.3. Efficacy

The efficacy of the CIP–Ca and CIP–Cu complexes was evaluated in vitro on planktonic bacteria by measurements of the minimum inhibitory concentrations (MICs). The MICs in CIP against *PA* and *Staphylococcus aureus* were not modified in the presence of a calcium concentration that allowed an 84% reduction in its P_app_ across a pulmonary epithelium model [23]. Similar results have been found with other metal ions, including cupric ions on planktonic PA01 [31]. However, the major challenge associated with therapy against chronic pulmonary infections in CF patients is the formation in the lung of biofilms, which leads to treatment-resistant infections. These biofilms, mainly present in the conductive area of the lungs [57], are aggregates of *PA* (50–100 μm large) entrapped in a self-produced matrix of anionic polymers (polysaccharides, proteins, eDNA, …), and surrounded by the patient’s thick mucus (also composed of anionic polymer) and polymorphonuclear leukocyte. One of the proposed mechanisms that contribute to the decrease in the susceptibility of *PA* to inhaled cationic antimicrobials, such as tobramycin, is the binding and sequestration of positively charged antibiotics by these anionic polymers [47,58,59,60]. The sustained pulmonary concentration of CIP in the presence copper is due to the formation of a positively charged complex (CIP–Cu)^2+^ that reduces the CIP P_app_ and lung–blood absorption rate. Thus, as for tobramycin, this complex could also be sequestered by the polymers forming the biofilms, which would decrease the efficacy of CIP. However, in a study where the efficacy of the CIP–Cu complex on *PA* biofilms was evaluated by time-kill experiments and measurement of the biomass of the biofilm [61], no difference was observed between the free CIP and CIP–Cu.

Two in vivo efficacy studies were performed in model rats that developed chronic pulmonary infection by the instillation of *PA*-loaded agar beads [55,62]. This lung infection model has been shown to mimic the chronic infection and inflammation that affects CF patients [63,64,65]. In one of the studies, the efficacy of the powder made of microparticles loaded with CIP–Cu was compared with that of a powder of micronized CIP–HCl [62]. Animals were dosed (6 mg/kg of CIP) on day 4 and day 6 post-infection with the dry powders in a nose-only inhalation exposure system (NOIES). CIP–Cu and CIP–HCl powders showed similar aerodynamic properties and comparable CIP lung deposition. However, treatment with CIP–Cu reduced by 4-log the number of CFU of *PA* per lung (day 8, end-point), whereas CIP–HCl’s effect was not different from the untreated control group.

It is unclear whether the high concentrations of antibiotics usually found in the sputum of nebulized CF patients, well above the MIC values, translate to more effective killing of *PA* [52]. These high concentrations could be due to the deposition of the antibiotics mainly in the large airways, which means that less drug would be available for the rest of the bronchial tree, especially for the less ventilated diseased areas, which could receive a lower dose of the drug than healthier regions of the lung. If this is the case, IV antibiotic therapy should result in more effective concentrations of antibiotics in diseased areas compared to inhalation therapy. Thus, a second study compared the efficacy of the CIP–Cu-loaded microparticles administered IT using a dry powder insufflator (DP-4, Penn-Century Inc., Philadelphia, PA, USA) with a group that received the same dose of CIP IV [55]. On day 8 after infection, the two groups were treated and the number of CFUs per lung was evaluated 4 and 24 h after treatment. The same reduction of 2 logs was observed for the two groups at time 4 h. However, after 24 h, the regrowth of bacteria at a number of CFUs per lung equivalent to the control was obtained for the group treated with IV, while a significant reduction of 1 log was still observed for the group that received the CIP–Cu microparticles IT.

#### 2.3.4. Safety

Lamy et al. studied the potential toxicity of CIP–Ca or CIP–Cu formulations by monitoring pulmonary integrity markers (Lactate dehydrogenase (LDH) and total protein concentration). No differences were observed between both formulations, a CIP solution of an equivalent dose after IT administration, and a negative saline control, suggesting that the integrity of the blood–lung barrier was preserved [22]. After IT administration of the CIP–Cu microparticles to healthy rats, the total plasma Cu^2+^ and Ca^2+^ concentrations measured between 0 and 18 h post-administration were constant (1.1 ± 0.06 and 99 ± 2 µg/mL, for Cu^2+^ and Ca^2+^, respectively). In ELF, the total Cu^2+^ concentration returned to physiological values within 18 h of IT administration. In contrast, the CIP concentrations in ELF were still higher than the MIC of CIP against most strains of *PA* [66]. This indicates that the dose of CIP, and therefore the dose of copper, may be decreased while effective concentrations of CIP may still be obtained. It also suggests that an effective dosing regimen could be found without the accumulation of copper in ELF. Additionally, bacterial infections attract macrophages and neutrophils to the lungs, which produce proteolytic enzymes that catalyze elastin degradation. The restoration of elastin increases the need for copper in the lungs to activate the enzymes responsible for its repair, such as the lysyl oxidase metalloenzymes. [67]. Similarly, COPD subjects have lower levels of copper than healthy subjects [68]. Co-administration of copper and CIP to COPD patients with lung infection may help fight infection and restore copper levels in these people. Likewise, copper-heparin inhalation therapy to repair emphysema has recently been investigated [67].

Copper has recently emerged as the focus of inorganic synthesis programs in which Cu complexes have been prepared for different indications, including viral infections, inflammatory diseases, and microbial infections [69]. However, copper inhalation should be envisaged cautiously, as it was found to be responsible for stimulated pulmonary collagen accumulation and fibrosis formation [70].

## 3. Control of the Appearance Rate of FQs in Pulmonary ELF: Controlled Dissolution and Controlled Release

Following deposition, the absorption rate of inhaled drugs is related to the epithelium membrane’s permeability, surface area, and drug concentration in mucosal fluids [71]. It is possible to control the concentration of the drug in mucosal fluids by controlling the rate of dissolution or release from a dry particle deposited in the lung. Accordingly, low water-soluble drugs that have limited lung absorption by their dissolution rate have a prolonged elimination half-life and increased average residence time in the lung [56]. For example, the poorly soluble antifungal agent amphotericin B can persist in the lungs for several days after inhalation [71]. Often, in this case, the plasma concentration profile of the drug is also changed after the inhalation of dry powder compared to after nebulization of a solution or IV administration. Mainly, the rate of drug elimination in plasma (*kel_(plasma)_*) is reduced due to the slow rate of absorption from the lung (*ka_(lung)_)*. This phenomenon is often called flip-flop kinetics. Indeed, since the drug cannot be eliminated until it is absorbed, the elimination phase of the drug profile reflects the input *ka_(lung)_*, rather than the output *kel_(plasma)_*. This causes the *ka_(lung)_* to be the rate-limiting step *(kel_(plasma)_ > ka_(lung)_*), making it slower and causing an increase in the plasma half-life. In the pulmonary mucosal fluids or ELF, the upward part of the drug concentration profile reflects the real ka (*k_release_* > ka*_(lung)_*) while the downward part of the curve becomes a reflection of the release/dissolution rate (ka*_(lung)_* > *k_release_*).

Guided by the flip-flop concept, efforts have been made to slow the rate of release or dissolution of FQs after inhalation in order to increase their residence time in the lungs. Two approaches, one using CIP–loaded liposomes and one using a CIP dry powder with low aqueous solubility and a slow dissolution rate, have been clinically evaluated.

### 3.1. Control of the Apparent Solubility of CIP in a DPI Formulation to Decrease its Dissolution Rate

In the controlled dissolution rate approach, the appearance of the drug in the surrounding medium is controlled mainly by the apparent solubility of the drug and the contact surface between the particle and the surrounding medium. The apparent solubility of the drug can be adjusted by various parameters, such as the nature of the solid state (polymorphs, amorphous, co-crystals, solvates, etc.), the formation of salts (nature of the counterion), or the use of adjuvant-like surfactants [72]. Very often, the particles formulated for DPI are prepared by spray drying and have an amorphous solid state with an increased apparent solubility compared to crystalline solids [2,73]. The total surface contact between a dispersed powder and its dissolution medium increases when the size of the particles decreases or their porosity increases. For pulmonary inhalation, particles of a unit density must have a geometry diameter < 5 µm in order to achieve effective delivery as an aerosol into the lungs (if spherical). This requirement for inhalation greatly increases the rate of dissolution of the powder for inhalation compared to powders formulated for oral administration. Likewise, particles that have a mass median aerodynamic diameter (MMAD) of around 5 µm and that deposit mainly in the respiratory tract will have a lower dissolution rate than particles with an MMAD of 1 µm, which mainly deposit in the alveolar space [5].

#### 3.1.1. Pharmaceutical Properties—Development

**Figure 3:**
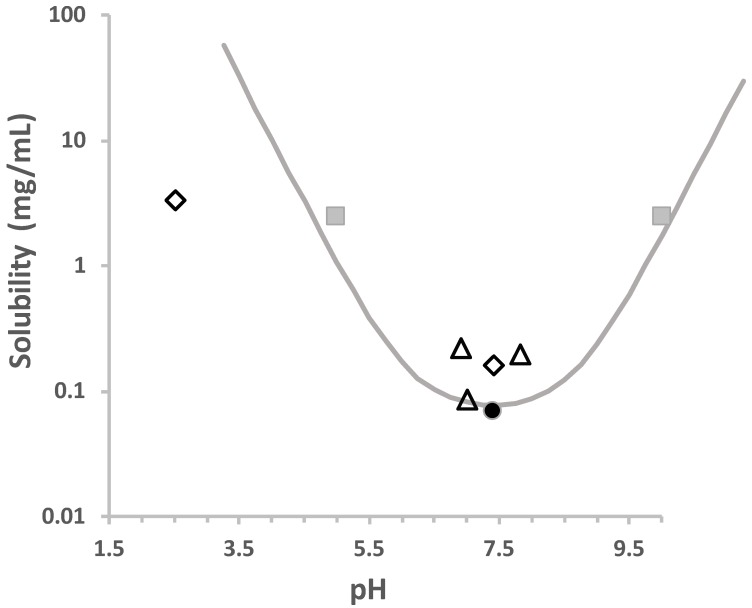
Effect of pH on CIP aqueous solubility calculated using the following equation: $S=S_{0}\left(1+10^{\left(pka_{1}-pH\right)}+10^{\left(pH-pka_{2}\right)}\right)$, with pka_1_ = 6.2, pka_2_ = 8.6 [20,75]. Individual values are from Yu et al. [76] (empty triangles); McShane et al. [20] (plain dot); Blokhina et al. [77] (empty diamond; Roca Jalil et al. [78] (square). Values were measured between 25 and 30 °C.

Bayer HealthCare in collaboration with Nektar Therapeutics and Novartis companies developed a CIP–based DPI as a therapy to reduce exacerbations due to respiratory bacterial pathogens (mainly *PA*) in adults with NCFB. The advantage of CIP–DPI over established liquid inhalation systems for antibiotics is the high and reproducible deposition of CIP in the lungs, the reduced application time (seconds compared to several minutes), and the reduced handling for the device compared to nebulizers (requiring cleaning and sterilization) [20]. This DPI combines three advances in the field of inhalation. First, the selection of the solid state of the CIP, crystallized in a neutral hydrate form (CIP·3.5 H_2_O) with low aqueous solubility and a slow dissolution rate, allows a favorable pulmonary PK (elimination rate from the lung controlled by the dissolution rate) [14,74]. CIP contains two ionizable groups, a carboxylic acid (pka1 = 6.2), and a piperazine group (pka2 = 8.6), conferring a pH-dependent solubility (Figure 3) [20,75]. In solution, at a pH two units lower than the pKa of the carboxylic acid (pH ~4), approximately 99% of the CIP molecules have a positive charge. At the physiological pH of the lungs (pH ~7.4), the carboxylic acid group is deprotonated, and CIP exists as a zwitterion (CIP^±^ with a net neutral charge). This zwitterion is often referred to as the betaine form, having a solubility around 70 µg/mL [20,74]. As pKa1 and pKa2 are separated by less than three units, CIP can also be present in the uncharged ampholytic form CIP^0^ [75]. The betaine form of CIP is the form used to formulate the CIP–DPI and its slower dissolution rate compared to CIP–HCl, the usually used solid salt form of CIP, increases its lung targeting. Indeed, A half-life of 13.5 h in rat lungs following oral inhalation has been demonstrated with the neutral form while CIP–HCl is rapidly absorbed into the systemic circulation, with a half-life in rat lungs of less than 1 h [14,20]. Additionally, following IT administration of a 7.5 mg/kg suspension, the AUC for the neutral form was increased 40-fold and the C_max_ is increased 8-fold in rat lungs relative to CIP–HCl [14].

The second advance in CIP–DPI is the use of the PulmoSphere ™ technology (Novartis, San Carlos, CA, USA) [79]. This formulation process produces a dry CIP powder with optimal aerodynamic properties, allowing the majority of the nominal dose to be deposited in the lungs (>50%). The low water solubility of the CIP betaine allowed its formulation using the PulmoSphere ™ technology. In this process, CIP betaine hydrate crystals are jet milled to obtain fine particles that are dispersed in the water phase of an oil-in-water emulsion [20,80]. After spray drying of the emulsion, crystals of CIP betaine are coated with a porous layer of phospholipids (1,2-distearoyl-sn-glycero-3-phosphocholine (DSPC)), reducing the cohesion forces between particles and making them easily dispersible. The highly porous particles contain 65% w/w of CIP and have a highly homogenous particle size distribution with a mass median aerodynamic diameter (MMAD) in the range of 1 to 5 μm [80,81]. These factors lead to reduced inter-patient variability in lung deposition from 30% to 50% for micronized drug blends to about 10% to 20% for PulmoSphere^TM^ formulations, requiring lower total doses to achieve comparable exposure [81].

The third advance is the use of a pocket-sized breath-actuated dry powder inhaler (T-326) that requires no special cleaning or disinfection. This device has a low-to-medium resistance (0.025 kPa^1/2^/L/min) and is already used in the USA and EU for the inhalation of tobramycin dry powder for the management of CF patients with *PA* (TOBI^®^ Podhaler^TM^) [82].

#### 3.1.2. Pharmacokinetics—Clinical Studies

A phase 1 PK study conducted using CIP–DPI was performed in six healthy male adult volunteers who inhaled a single 32 mg dose of CIP [13]. Approximately 40% of the total dose reached the lung. The terminal plasma elimination half-life was 9.5 h, i.e., longer than previously reported with oral or parenteral formulations, suggesting that elimination from the respiratory tract was prolonged (flip-flop effect). In a follow-up dose escalation (32.5 or 65 mg) phase I study conducted in adult CF patients, low systemic exposure and high but variable sputum exposure were observed [12]. A study performed on COPD patients showed similar PK data [83]. The 32.5 mg twice daily dosing regimen has thus been selected as the best dosage for further development in NCFB patients. Indeed, this dosage provided high drug concentrations in the sputum, low systemic exposure, and good tolerability. In NCFB patients, CIP–DPI inhalation resulted in CIP sputum C_max_ around 58-fold higher than those generally attained by systemic administration of therapeutic doses. Additionally, unbound CIP plasma C_max_ was around 24-fold lower than that usually achieved by systemic administration [84]. Accordingly, CIP C_max_ was 1400-fold higher in the sputum than in the serum after CIP–DPI inhalation in NCFB patients.

#### 3.1.3. Efficacy

In a phase 2 randomized placebo-controlled multicenter study, CIP DPI 32.5 mg twice daily for 28 days was evaluated for its capacity to reduce the total sputum bacterial load in NCFB patients. Various bacterial species were considered, including *PA*, *Staphylococcus aureus*, *Streptococcus pneumoniae*, *Haemophilus influenzae*, and *Klebsiella pneumoniae*. After 28 days, a significant reduction in the bacterial load of −3.62 log10 CFU/g of sputum was obtained with CIP–DPI compared to a reduction of −0.27 log10 CFU/g of sputum for the placebo [84,85]. The maximum reduction in the number of bacteria was reached on days 7 to 9 and was maintained for the remainder of the 28-day period. Unfortunately, regrowth of the bacteria during the 28-day period without treatment was observed in most patients, and 8 weeks after the end of treatment, the average bacterial load in the sputum was similar between the CIP–DPI and placebo groups.

Then, two phase-3 clinical studies (RESPIRE 1 and 2) aimed at establishing the efficacy of CIP–DPI in the treatment of chronic infections in NCFB patients were carried out [86,87,88]. The measures of efficacy included the following four endpoints: The first criterion was the increase in time until the first exacerbation (primary endpoint); the second was the reduction in the frequency of exacerbations; the third was the improvement in patient-reported outcomes on lung functions; and the fourth was the improvement in the eradication of bacteria initially present and a decrease in the acquisition of new pathogens. Two dose regimens of CIP–DPI of 14-day on/off and 28-day on/off were evaluated. While the results for the CIP 14-day regimen in RESPIRE 1 demonstrate an effect of a prolongation of the time to the first exacerbation, the FDA advisory committee had reservations about the overall treatment benefit in NCFB patients based on the results of the various endpoints measured. The committee pointed out that the results for the 14-day regimen were not consistent between the two trials, with a significant treatment effect on the time to the first exacerbation in RESPIRE 1, which was not reproduced in RESPIRE 2. The 28-day regimen did not meet the primary endpoint in either trial [89]. Citing inconsistent data, concerns about drug resistance, and an ability to meet only one of four primary endpoints in two studies, a majority of the FDA Antimicrobial Drugs Advisory Committee voted not to recommend CIP DPI for the treatment of the NCFB [89].

#### 3.1.4. Safety

Following oral inhalation of a dry powder containing 32.5 mg CIP, the mean CIP plasma C_max_ in healthy subjects, COPD, and bronchiectasis patients ranged from 0.10 to 0.13 mg/L, which is at least 10-fold lower than the C_max_ following oral and intravenous administration of CIP at approved clinical doses [83]. Due to the low systemic exposure of CIP following oral inhalation of CIP dry powder, no dose adjustment is warranted for patients with hepatic or renal impairment and patients taking concomitant medications.

### 3.2. Control of the FQ Release Rate Using Particulate Systems

In the controlled release approach, the drug is trapped in a particle whose properties control the rate of drug appearance as a solubilized molecule in the surrounding environment. For example, drugs can be dispersed or dissolved in particles made of biodegradable polymers, such as polyesters (poly(lactic acid-glycol) (PLGA), poly(lactic acid) (PLA), and poly(caprolactone) (PCL)…), whose degradation rate will control, along with other factors, the drug release rate [2,90]. Due to the limited drug loading capacity of these systems relative to the high dose of antibiotics needed to kill bacteria, repeat administrations (perhaps once or twice a week) should still be required with these systems. On the other hand, it is important in the treatment of chronic infections by repeated pulmonary administration to avoid the accumulation of polymer in the lungs. Thus, relatively fast degrading polymers should be used in this strategy. This can be done with PLGAs by selecting low molecular weight fully amorphous polymers or polymers with a large proportion of the glycolide monomer to increase the hydrophilicity of PLGA and its rate of hydrolysis. A controlled release of a drug can also be obtained using liposomes [2,19]. These systems have a relatively high loading capacity and are made up of endogenous compounds found in the ELF. Their stability and drug release rate depend on the fluidity of their membrane, which can be adjusted by incorporating sterols (cholesterol) or by selecting the chain length of the fatty acids constituting their phospholipids.

An interesting property of these two systems is that they can be designed to be taken up into pulmonary cells or, on the other hand, to avoid macrophage phagocytosis. For example, large porous particles (~10–15 μm) have demonstrated s effective lung deposition and enhanced lung residence as a result of their large diameter and reduced clearance by macrophages in comparison to small microparticles [2]. This difference is important when treating either extracellular or intracellular infections.

#### 3.2.1. Control of the Release Rate Using Polymer Microparticles

Biodegradable polymer drug carriers are an attractive option for lung delivery as they allow for drug-sustained release and specific targeting. Several studies have shown the possibility of loading CIP into PLGA particles. PLGA is a biocompatible and biodegradable polymer and has been widely used in the formulation of sustained release particles for inhaled therapies [91]. The FQ-loaded PLGA particles are usually prepared by the double emulsion solvent evaporation method. In a study, Thomas N. et al. [92] formulated microparticles (12 μm) and nanoparticles (300 nm) using this method, which contained CIP charges of 7.3% and 4.5% (w/w), respectively. The drug release profiles were comparable for both particle sizes. The in vitro release profiles started with a burst effect during the first 24 h with about 50% of the CIP released, followed by a sustained drug release through zero-order kinetics between 1 and 4 days, and complete release was observed within 5 days. The burst release is explained by the presence of loosely bound fraction of the drug at the particle surface. The sustained release reflects the combination of drug diffusion within the polymer matrix and the progressing erosion of the polymer [93]. The efficacy against *PA* or *Staphylococcus aureus* bacterial strains was also studied and no difference was observed between the MIC of CIP or CIP–loaded microparticles after 22 h, where almost 70% of the CIP was released according to the release study. The antibiofilm performance of the treatment was also evaluated. Over 6 days, the CIP–loaded PLGA micro- or nanoparticles had the same complete eradication on the *PA* bacteria biofilm. For *Staphylococcus aureus*, the performance of the microparticles was lower than the free drug.

Similar results were obtained by Jeong Y. et al., where CIP–encapsulated PLGA nanoparticles were obtained with a 100–300 nm size, with around 4.5% drug loads. The drug release study showed an initial burst effect for 12 h (with approximately 20% of the CIP released) and then a continuous release for 2 weeks [94]. This burst release permitted a high initial antibiotic concentration followed by an extended release. This release profile could be an advantage for the successful eradication of the bacteria biofilm, where a high initial antibiotic exposure would minimize the antibiotic tolerance of the surviving bacteria cells [95]. Susceptibility testing of CIP–loaded nanoparticles against *E. coli* was realized in vitro and in vivo. The in vitro results showed a similar activity of the encapsulated CIP compared to the free CIP. For the in vivo experiment, an *E. coli*-containing dialysis membrane was implanted into the peritoneal cavity of mice. A subcutaneous single dose of CIP (25 mg/kg) failed to control the bacterial growth while the CIP–loaded nanoparticles helped to significantly reduce the growth of bacteria due to the sustained release [94].

To date, no PK have been realized with CIP–loaded PLGA particles in the lungs. LVX-loaded PLGA particles have also been studied in order to decrease the absorption rate in the lung. Cheow et al. [96] reported two methods for the preparation of LVX-loaded polymer particles: A nanoprecipitation (NPC) method was first employed to produce the burst release profile of LVX-loaded nanoparticles, where LVX is predominantly absorbed on the nanoparticle surface. A second method using an emulsification-solvent-evaporation (ESE) method was employed to prepare LVX-loaded nanoparticles with a biphasic release profile. The NPC method produced smaller particles (100 nm) than the ESE (200 nm). The drug loading was low due to LVX’s high water solubility (around 1%). LVX-loaded PLGA nanoparticles prepared by the NPC method exhibited monophasic burst release profiles (80% of the loaded drug was released in 1 h) and the entire drug was released after 6 h. Nanoparticles produced by the ESE method exhibited biphasic extended release profiles (80% of the loaded drug is released in 1 day) followed by a slower release, resulting in the entire drug being released after only 6 days. Efficacy testing against an *E. coli* biofilm showed similar results for LVX or LVX-loaded nanoparticles [96].

Gaspar et al. [16] produced 5-µm LVX-loaded PLGA microspheres with a 10% drug load. The release profile was characterized by a burst effect of 40% of the LVX within the first 30 min followed by a sustained release up to 72 h, where around 75% of the LVX was released. PK studies were conducted in rats after IT aerosolization of the LVX-loaded microspheres. For comparison, LVX as a solution or as immediate-release chitosan microspheres were also administered. The concentration profiles in the plasma and in the ELF were identical after IT administration or IV LVX administration, as expected with high-permeability drugs. As anticipated from the in vitro release studies, the chitosan microspheres released LVX very rapidly and the PK of LVX in ELF and plasma were similar to the PK obtained after IT administration of an LVX solution. For these two formulations (solution and immediate-release chitosan microsphere dry powder), the LVX diffused almost instantaneously through the broncho-alveolar barrier and the ELF-to-unbound plasma AUC ratios were slightly above 2. In contrast, after IT administration of PLGA microspheres, a high ELF-to-unbound plasma AUC ratio (311) was observed and high LVX concentrations were maintained in ELF for at least 72 h, showing the successful sustained release of LVX by PLGA microparticles.

Another advantage of the encapsulation is the possibility of combined therapy, which would help to reduce bacterial resistance by taking advantage of the different mechanisms of action. However, only a few studies have been published on this topic for FQs. For instance, moxifloxacine/rifampicin or CIP/minocycline/metronidazole slow-release microparticles have been successfully made and showed a slow release profile and similar antibacterial activity to free drugs [97,98].

#### 3.2.2. Control of the Release Rate of CIP Using Liposomes

Liposomes are phospholipid-based vesicles that are very similar to lung surfactants in mammals and are therefore biocompatible and biodegradable in the lungs. There is considerable interest in liposomes for pulmonary delivery due to their ability to trap a large amount of drug with different physicochemical properties. This increases the apparent solubility of the drug and helps control the rate of its appearance in the body (reduces irritation and side effects, reduces degradation, control of the absorption rate). Thus, after inhalation, a liposome can maintain the drug in the pulmonary system for an extended period. For the pulmonary delivery of antimicrobials, liposomes also have the advantages of improving their diffusion through microbial biofilms and increasing their uptake by macrophages. Thanks to all these benefits, Arikace^®^, a liposomal formulation of amikacin specifically labelled for delivery by inhalation, was approved in 2018 in the USA for use as part of a combination antibacterial drug regimen against *Mycobacterium avium complex* [99]. Preclinical studies have shown that this liposomal formulation of amikacin facilitates lung delivery, penetration into *Mycobacterium* biofilms, and alveolar macrophage uptake compared to free amikacin [100]. Because of the rapid clearance of FQs from the lungs following pulmonary administration, several liposomal formulations have been developed. The most advanced is a liposomal formulation of CIP for inhalation branded Linhaliq^®^ in Europe and Apulmiq^®^ in the USA (previously called Pulmaquin^®^), which is now in the final stages of clinical testing [17,101]. Some good recent reviews [19,102,103] describe their development in detail and will therefore only be discussed briefly here: Linhaliq^®^ is a 80–100 nm liposome-encapsulated CIP delivered once daily through a jet nebulizer (PARI LC^®^). The liposomes are a mix of hydrogenated soy phosphatidylcholine and cholesterol, with a lipid/drug ratio of 2. Linhaliq^®^ is a 1:1 mixture of liposome-encapsulated CIP and free CIP. This formulation allows an immediate effective dose from the free component (bust effect) and a sustained delivery over 24 h from the liposome-encapsulated component. Once daily inhaled Linhaliq^®^ was tested in the ORBIT-2 (Once-daily Respiratory Bronchiectasis Inhalation Treatment) 168-day multicenter international phase 2b clinical trial in 42 adult patients with NCFB. After 28 days of treatment, a significant mean reduction of 4.2 log10 CFU of *PA* in the sputum in the Linhaliq^®^ group was observed versus a very small mean decrease of 0.1 log10 units in the placebo group. Linhaliq^®^ was well tolerated and there were no significant decreases in lung function [19]. The safety and efficacy of inhaled liposomal CIP (ARD-3150) were evaluated in two phase 3 trials in NCFB patients (ORBIT -3 and 4). Inconsistent results were obtained, where ORBIT-4 but not ORBIT-3 showed a significant longer time to the first pulmonary exacerbation (230 vs. 163 in the placebo group) and a lower rate of exacerbations (37% reduction) compared to the placebo [101,104]. These results are encouraging and further experiments are needed to evaluate the efficacy in other target groups of patients (CF patients, Q fever patients, intracellular microbial agents) or a better duration of treatment [105,106].

## 4. Conclusions

A significant limitation of locally delivered treatments for chronic pulmonary infections with a new pharmacological class of antimicrobials is often their short residence time within the airways after nebulization. FQs, such as CIP, for example, undergo rapid absorption from the airway lumen, which limits their benefit for pulmonary administration. Two main strategies were investigated up to clinical trials to increase the residence time of FQs after inhalation: The controlled permeability strategy consists in decreasing their lung apparent permeability and systemic absorption rate by complexation with multivalent metal cations. This approach, which was developed with metal cations, could also be envisaged with other forms of interaction that transiently increase the apparent polarity of the drug, such as complexation with cyclodextrins or the formation of polar ion pairs [107,108]. The second, the controlled release strategy, involves controlling the rate of FQ release from inhaled solid or soft particles. This strategy can be developed by inhaling a low-soluble form of solid FQ or by encapsulating FQ in a particulate system, such as PLGA microparticles or liposomes. Both strategies showed an important increase in the FQs’ residence time (50- to 600-fold) in animals and humans in various clinical trials on patients with different diseases, such as CF, COPD, or NCFB, and seem promising. However, only one, the LVX-Mg complex of the Quinsair^®^ formulation, is actually on the market. Even if the two approaches can lead to a significant increase in the pulmonary residence time for molecules with a relatively high apparent permeability, they present certain notable distinctions. In the controlled permeability system, the drug is already in solution and then is immediately active against the extracellular bacteria, which may be an advantage over the controlled release system, where the release rate has to be finely tuned to be the most efficient. On the other hand, the controlled release system involves the use of particles that can concentrate in the macrophages. This is an advantage if the targeted bacteria are developing intracellularly, such as the case for tuberculosis or tularaemia. However, this could be a drawback for the treatment of extracellular bacteria, such as *PA*. Another interesting point for the authors is the possibility offered by the controlled release strategy of co-releasing at the same time as FQ adjuvants that increase their effectiveness. For example, the incorporation of farnesol, a natural quorum sensor inhibitor, and CIP in a liposomal formulation exhibited a very interesting outcome. The minimum biofilm eradicating concentration (MBEC) value against PA01 obtained using the co-delivery system was reported at 0.128 μg/mL of CIP, essentially the same as its reported MIC value against planktonic bacteria [109]. Finally, despite disappointing clinical trials (ORBIT and RESPIRE), the controlled release strategy is still encouraging but needs further developments. High lung permeability FQs are a good example of the application of these strategies but other treatments with high lung permeability antibiotics, such as chloramphenicol, thiamphenicol, rifampicin, or linezolid, could also benefit from these strategies.

Controlling the lung residence time of FQs is of course not limited to the strategies developed in this manuscript. Several other strategies to control drug permeability or release are under development. Non-particulate approaches could use macromolecules to increase the apparent molecular weight of the drug and then decrease its apparent permeability. For instance, cyclodextrin/rifampicin complexes have been studied in vitro [107]. In this study, the first objective was to increase the apparent solubility of rifampicin using cyclodextrin, but it appeared that the apparent permeability of rifampicin evaluated with Calu-3 cells was 40% lower when it was complexed with cyclodextrins, and that cyclodextrins could be used in the lungs to increase its residence time. Similarly, LVX, ofloxacin, or moxifloxacin/cyclodextrin complexes have been studied by other authors as a controlled release strategy [110]. Pegylation may also be used to increase the apparent size of the drug and decrease its permeability through biological barriers. Pegylated tobramycin has been successfully developed to increase mucus penetration and antibiofilm efficacy [59]. Although the P_app_ of this molecule has not been evaluated through models of the pulmonary epithelium, it has been shown that pegylation reduces pulmonary permeability and that this decrease increases with the size of the PEG molecule [111]. The synthesis of a prodrug is usually aimed at increasing the drug bioavailability after oral administration. However, the opposite would be an interesting strategy to decrease the drug permeability for pulmonary administration. A better knowledge of enzymes specific to the lung would be needed to control the release rate of such a prodrug. An antibiotic prodrug could be designed to target specific bacteria if the linker (ester, amide, disulphide) used in the conception of the prodrug is a specific substrate for a bacterial enzyme. There are examples for CIP or pyraminazide using such an ester linker (for a review, see [112]). Similarly, prodrugs could be designed with a bacteria-targeted moiety linked to the antibiotic. This targeting moiety could be an antibody directed against a molecule of the bacterial wall or even another antibiotic targeting the bacterial wall for a synergistic approach (for a review, see [113]). The particulate approach is even richer since the decoration of the nano- or microparticles may target a specific environment (mucus, biofilm) or bacteria. One interesting example is the ROS-responsive nanoparticles developed by Wang et al. In these nanoparticles, moxifloxacin is complexed with phenylboric ester-modified cyclodextrins. This ester link is cleaved in the presence of a low H_2_O_2_ concentration, which is produced by neutrophils at the site of infection. This original controlled-release formulation is thus able to deliver moxifloxacin specifically to infected tissue [114]. With the increasing bacterial resistance to antibiotics and the lack of new antibiotics, the development of these new strategies is increasingly necessary.

## Figures and Tables

**Table 1:** Apparent association constant (Ka) between FQ and cations.

	CIP–Cations (M^−1^, 1:1 Complexes) [31]	LVX-cation (M^−2^, 2:1 Complexes) [32]
Ca^2+^	100	562
Fe^2+^	122 *	34,673
Mg^2+^	720	4898
Fe^3+^	1551 *	
Zn^2+^	2700	27,542
Al^3+^	88,000	
Cu^2+^	906,900	

* unpublished data.

**Table 2:** FQ–cation apparent association constant ranking.

ciprofloxacin	Cu^2+^ > Al^3+^ > Zn^2+^ ≈ Fe^3+^ > Mg^2+^ > Fe^2+^ ≈ Ca^2+^	[31]
	Al^3+^ > Fe^3+^ ≈ Cu^2+^ > Zn^2+^ > Mg^2+^	[33]
	Al^3+^ ≈ Fe^3+^ > Zn^2+^ ≈ Mn^2+^ ≈ Mg^2+^	[34]
levofloxacin	Fe^3+^ > Fe^2+^ > Mg^2+^ ≈ Ca^2+^	*
	Al^3+^ > Fe^2+^ ≈ Zn^2+^ > Mg^2+^ > Ca^2+^	[32]
	Al^3+^ ≈ Cu^2+^ > Fe^3+^ > Mg^2+^	[33]
enrofloxacin	Al^3+^ > Fe^3+^ ≈ Cu^2+^ > Mg^2+^	[33]
sparfloxacin	Al^3+^ > Cu^2+^ > Zn^2+^ > Mg^2+^	[33]
moxifloxacin	Fe^3+^ > Mg^2+^ ≈ Fe^2+^ ≈ Ca^2+^	*
pefloxacin	Fe^3+^ ≈ Mg^2+^ > Fe^2+^ ≈ Ca^2+^	*

* unpublished data.

**Table 3:** Estimates of LVX C_max_ and AUC_0-24_ in sputum of CF patients expressed as a number of times those were calculated in the serum obtained from the phase 2 and phase 3 clinical studies.

Clinical Study	LVX Sputum Versus Serum C_max_	LVX Sputum Versus Serum AUC_0-24_
MPEX-204	5000 to 7000-fold higher in sputum than in serum	300-fold higher in sputum than in serum
MPEX-207	3500-fold higher in sputum than in serum	400-fold higher in sputum than in serum
MPEX-209	1700-fold higher in sputum than in serum	600-fold higher in sputum than in serum

**Table 4:** The relationship between the affinity of the complex formed between CIP and the metal cation and pulmonary exposure to CIP after IT administration of the complex.

	(CIP–Ca)^2+^	(CIP–Cu)^2+^	CIP solution
Ka (M^−1^) (from [31])	100	906,900	
Concentration (µM) needed to reduce the CIP P_app_ by 50% (from [31])	40	0.04	
ELF **C_max_** (µg/mL) after IT administration to rats (from [22])	26.5 ± 17.7	142.3 ± 81.5	1.0 ± 0.4
CIP AUC_ELF_ to AUC_plasma_ ratio after IT administration to rats (from [22])	203	1069	9.8

## Abbreviations

AUC	area under the concentration-time curve
BAL	broncho-alveolar lavage
BID	twice daily
CF	cystic fibrosis
CFU	colony forming unity
CIP	ciprofloxacin
C_max_	maximum concentration
COPD	chronic obstructive pulmonary disease
DPI	dry powder inhaler
ELF	epithelial lining fluid
EMA	European medicine agency
ESE	emulsification-solvent-evaporation
FDA	food and drug administration
FQ	fluoroquinolone
IT	intratracheal
IV	intravenous
Ka	association constant
*Ka_(lung)_*	absorption rate constant from the lung
*Kel_(plasma)_*	elimination rate constant from the plasma
LDH	lactate dehydrogenase
LVX	levofloxacin
MIC	minimum inhibitory concentration
NCFB	non-CF bronchiectasis
*PA*	*Pseudomonas aeruginosa*
P_app_	apparent permeability
PK	pharmacokinetics
PLGA	poly(lactic-co-glycolic acid)
QD	once daily
TIS	tobramycin solution for inhalation
